# PFOA Damages Blood–Testis Barrier Integrity in Mice by Inhibited Glycolysis Caused H3K18 Lactylation Modification Impairment

**DOI:** 10.3390/toxics14050399

**Published:** 2026-05-07

**Authors:** Zhengqi Song, Jinxin Ruan, Lingqiao Wang, Ke Cui, Zhiling Wu, Weiyan Chen, Yao Tan, Yiqi Wang, Guanghui Zhang, Guowei Zhang, Wenbin Liu, Zhiliang Cheng, Jun Li, Ziyuan Zhou

**Affiliations:** 1 The Key Laboratory of Environmental Pollution Monitoring and Disease Control, Ministry of Education, School of Public Heath, Guizhou Medical University, Guiyang 550025, China; 2023110040845@stu.gmc.edu.cn (Z.S.); 2023110040889@stu.gmc.edu.cn (J.R.); 2 Department of Environmental Health, College of Preventive Medicine, Third Military Medical University (Army Medical University), Chongqing 400038, China; wanglingqiao@tmmu.edu.cn (L.W.); cuike777777@163.com (K.C.); w84742914@126.com (Z.W.); weiyanchen@tmmu.edu.cn (W.C.); xiaoyue7122@163.com (Y.T.); wangyiqi@tmmu.edu.cn (Y.W.); zhgh221@tmmu.edu.cn (G.Z.); zhangguowei.2009@163.com (G.Z.); liuwenbin@tmmu.edu.cn (W.L.); 3 School of Chemistry and Chemical Engineering, Chongqing University of Technology, Chongqing 400054, China; purper@cqut.edu.cn

**Keywords:** perfluorooctanoic acid, blood-testis barrier, glycolysis, histone lactylation

## Abstract

The molecular mechanism underlying male reproductive toxicity associated with Perfluorooctanoic acid (PFOA), a persistent environmental endocrine disruptor (EDC), has not yet been fully elucidated. Six-week-old male C57BL/6 mice were treated with PFOA by oral gavage at 0, 1.25, 5, 10, and 20 mg/kg/day for 35 days to explore its toxic effects on the male reproductive system and the underlying mechanisms. Analyses of semen quality, testicular histopathology, and blood–testis barrier (BTB) integrity revealed that PFOA caused dose-dependent structural and functional damage to the BTB, leading to markedly reduced semen quality. Based on transcriptomic sequencing and differential gene enrichment analysis, the glycolytic pathway was identified as a key regulatory target for PFOA-induced damage to the reproductive system. Further validation revealed that PFOA exposure inhibited glycolysis-related enzymes (Hexokinase 1 (HK1), Glucose Transporter 1 (GLUT1), and Lactate Dehydrogenase A (LDHA)), reduced lactate production and ATP synthesis, lowered Pan-Kla and H3K18la levels, and diminished H3K18la enrichment at the *Hk1*, *Glut1*, and *Ldha* promoters, whereas exogenous sodium lactate reversed these changes. This study is the first to identify the “glycolysis–lactate–H3K18la” chain as a key regulator in PFOA-induced BTB damage and spermatogenesis impairment, offering a new theoretical foundation for understanding EDC-induced male reproductive toxicity.

## 1. Introduction

Male reproductive health has become a central issue for global public health. Against the backdrop of a significant decline in global fertility rates, male infertility has been identified as one of the main contributing factors [1]. Semen quality, a key indicator of male fertility, is showing an increasingly alarming global decline. Sperm count continues to decrease at an average annual rate of 3.12%, while sperm motility and the percentage of normally shaped sperm are also deteriorating at the same rate [2,3,4]. Exposure to environmental endocrine disruptors (EDCs) has become one of the primary causes contributing to the decline in semen quality. Perfluorooctanoic acid (PFOA) is a typical EDC broadly used owing to its exceptional chemical and thermal stability as well as superior hydrophobic and oleophobic properties. It serves as an essential industrial additive for fluoropolymer manufacturing, and is commonly applied in non-stick cookware, waterproof textiles, food packaging and firefighting foam [5,6,7,8,9]. Emerging evidence confirmed that perfluoroalkyl and polyfluoroalkyl substances (PFAS) exposure disrupts seminal redox homeostasis, reduces antioxidant capacity and increases lipoperoxide levels. Such biochemical abnormalities occur prior to obvious DNA damage, acting as early biomarkers for male reproductive injury [10].

Epidemiological survey, based on 1206 men, demonstrated that logistic regression analysis revealed a significant positive association between seminal PFOA levels and the risk of asthenospermia (*p* < 0.05) [11]. Blood PFOA concentrations among residents in highly polluted areas can reach 92.03 μg/mL, far exceeding the global average of 4 ng/mL. PFOA levels in their semen are approximately 0.67 ± 0.908 ng/mL, and the decline in semen quality is even more pronounced [12,13,14]. This clinical evidence suggests that exposure to PFOA has become a significant environmental hazard factor for male reproductive health.

Sertoli cells are essential for maintaining male reproductive function, as they envelop and support germ cells at all stages, providing the necessary microenvironment for spermatogenesis. Their intercellular junctions also form the blood–testis barrier (BTB), which consists primarily of tight junctions (TJs), gap junctions (GJs), and adherens junctions (AJs). The BTB not only effectively maintains the structural and functional integrity of the testis but also provides an immunoprivileged environment for germ cells, ensuring their normal survival and orderly development [15]. Regulation of glucose metabolism by sertoli cells is key for ensuring male reproductive function. Unlike most normal cells in the body, which primarily rely on aerobic oxidation for energy under aerobic conditions, the energy metabolism of testicular sertoli cells is unique in that these cells primarily rely on glycolysis for energy in a relatively hypoxic environment (similar to the Warburg effect) [16]. The structural integrity and functional homeostasis of the BTB complex depend strongly on the energy and metabolic products generated via glycolysis in sertoli cells. Glycolysis-derived ATP serves as the key energy source for cells to maintain transmembrane ion gradients and acts as a key energy source for the localization, stabilization, and functional presentation of tight junction proteins (Zonula Occludens-1 (ZO-1), OCCLUDIN, and CLAUDIN) [17]. ATP shortage disrupts the cytoskeleton’s dynamic reorganization, essential for tight junction integrity, causing direct damage to the BTB [18]. In addition, lactate not only serves as the primary energy substrate for germ cells but is also involved as a signaling molecule involved in regulating the expression of BTB-related genes [19]. Inhibition of glycolysis leads directly to insufficient lactate production and energy deficiency, which in turn triggers the collapse of the BTB structure and functional disruption, ultimately exacerbating male reproductive damage [20,21]. Previous studies have demonstrated that exposure to PFOA can disturb overall glucose homeostasis in the body and induce metabolic disorders [22,23,24]. However, existing research has mainly focused on metabolic organs such as the liver, and it remains unclear whether PFOA specifically targets and inhibits glycolysis in the reproductive system.

Histone H3 lysine 18 lactylation (H3K18la) is a transcription-activating histone modification involved in the fine regulation of spermatogenesis, and its aberrant expression can lead to abnormal sperm morphology and reduced fertility [25,26,27]. H3K18la is stably expressed in sertoli cells, where it mediates widespread apoptosis [28]. While H3K18la is known to be involved in regulating male reproductive functions, its exact role in PFOA-induced sertoli cell dysfunction, BTB impairment, and spermatogenic issues is still not well understood. It is particularly important to investigate whether PFOA exposure influences BTB integrity through the regulation of glycolysis-related gene expression mediated by H3K18la, given that lactate, the end product of glycolysis, is the primary substrate for histone lactylation. Moreover, it is still unclear if external lactate can counteract the injuries mentioned earlier. Filling these research gaps is crucial for comprehending the reproductive toxicity mechanisms of PFOA and finding possible interventions.

Based on the above, in this study we established in vivo models of male mice exposed to PFOA and in vitro cellular models to (1) verify the toxic effects of PFOA exposure on BTB integrity and the resulting decline in sperm quality; (2) reveal the key role of glycolysis inhibition in PFOA-induced BTB damage; (3) explore the mechanism through which H3K18la modification regulates the expression of glycolysis-related genes in response to PFOA; and (4) confirm the reversal of PFOA-induced reproductive damage via exogenous lactate supplementation. This study not only improves our understanding of the reproductive toxicity of environmental EDCs, but also suggests that supplementation with lactic acid or the regulation of H3K18la levels may serve as potential targeted strategies for mitigating the reproductive toxicity of PFOA.

## 2. Materials and Methods

### 2.1. Establishing Animal Models of PFOA Toxicity

This study used 6-week-old male C57BL/6 mice (Beijing Weitong Lihua Co., Ltd., Beijing, China). In male C57BL/6 mice, puberty begins at 5–6 weeks of age, and 6 weeks constitutes a critical developmental stage of puberty [29,30]. The mice were kept under controlled conditions with a temperature of 20–26 °C, a relative humidity of 45–60%, and a 12 h light-dark cycle. Mice were allowed to freely consume standard chow and drink purified water, and their cages were lined with ample bedding to ensure normal activity. After one week of adaptation, the mice were randomly assigned into 5 groups (*n* = 20 per group): The control group was gavaged with ddH_2_O; the treatment groups were administered 1.25, 5, 10, and 20 mg/kg/day of PFOA (Sigma-Aldrich, St. Louis, MO, USA) (dissolved in ddH_2_O), respectively. The dosage volume was determined based on body weight; the gavage volume was 0.1 mL per 10 g body weight, administered continuously for 35 days (covering the entire spermatogenic cycle of the mice), with body weight monitored weekly. All animal experiments were approved by the Animal Welfare and Ethics Committee of the Army Medical University (AMUWEC20226243).

### 2.2. Animal Disposal and Sample Collection

Following gavage, male mice in each group underwent a 1-week washout period, after which they were housed with untreated female mice at a 1:2 ratio for a 2-week mating trial. After the breeding period, we collected blood samples from the orbital sinuses of each mouse (*n* = 10 mice per group) and euthanized the mice by cervical dislocation. Following necropsy, the testes and epididymis were dissected and weighed, and the testis index and epididymis index were calculated. One testis was rapidly frozen in liquid nitrogen and stored at −80 °C, while the other was fixed in 4% paraformaldehyde for subsequent relevant tests and experiments. Meanwhile, the remaining mice (*n* = 10 mice per group) were used for mouse neurobehavioral tests.

### 2.3. Sperm Motility and Count

Place the bilateral epididymides of the mouse in 1 mL of DMEM/F12 medium containing 0.1% BSA at 37 °C. Mince them using sterile ophthalmic scissors, then gently pipette to mix thoroughly. Incubate on a 37 °C heating plate for 5 min to promote sperm release, then prepare a sperm suspension. Dispense 5 μL of this suspension onto a matched commercial Suijia disposable sperm counting slide. Sperm quality parameters were analyzed using a Suijia SSA-II Plus computer-assisted sperm analysis (CASA) system (Suijia Biotechnology, Beijing, China). The analysis was performed at a frame rate of 60 Hz with 45 acquisition frames. The minimum contrast was set to 50, the minimum cell size to 5 pixels, and cell intensity to 90. The path velocity threshold was 10.0 μm/s, and the straightness threshold was defined as 0 [31]. For each sample, at least 6 random microscopic fields were captured, and no fewer than 500 valid spermatozoa were analyzed. The detected indicators included sperm count, progressive motility percentage and total motility percentage, all of which were automatically analyzed and calculated by the built-in software of the CASA system.

### 2.4. Sperm Abnormality Testing

Prepare a semen smear using 10 μL of sperm suspension. Allow the smear to air dry, then fix it with 95% ethanol. Stain with eosin staining solution, rinse under running water after staining, and allow to air dry. Observe sperm morphology in different fields of view under an optical microscope. Examine 500 sperm from each mouse, with three mice per group (Random sampling was performed from 10 experimental animals per group). When observing the final field of view, count all sperm within the field even if the 500-sperm limit has been reached, and calculate and record the sperm abnormality rate.

### 2.5. Hematoxylin-Eosin (H&E) Staining

Fresh testes were fixed in 4% paraformaldehyde, dehydrated, embedded in paraffin, and sectioned at 4 μm. After dewaxing and rehydration, sections were stained with H&E for histological observation as previously reported [12]. Spermatogenic epithelial structure and progressive maturation of germinal cells were assessed via the Johnsen histological grading scale ranging from 1 to 10 (Table 1). Fifty intact seminiferous tubules from each unilateral testis of individual mouse were evaluated, and the mean Johnsen score (MJS) was calculated and noted [32,33]. In addition, tubular differentiation index (TDI) and spermiogenesis index (SPI) were determined through histological quantification of 20 seminiferous tubules across 3 testicular tissue sections. Under light microscopy at 400× and 1000× magnifications, we quantified the proportion of seminiferous tubules possessing 3 to 4 layers of germ cells for TDI, together with those displaying complete spermiogenesis for SPI evaluation [34].

### 2.6. Immunohistochemistry of Testicular Tissue

The prepared testicular tissue sections underwent high-pressure antigen retrieval. After washing with PBS, endogenous peroxidase activity was blocked, and the sections were treated with goat serum for blocking, followed by incubation with anti-SOX9 (HY-P80335, 1:200, MCE, Monmouth Junction, NJ, USA) at 4 °C overnight. The next day, incubated with HRP anti-rabbit at room temperature for 2 h, and then observed under a microscope to capture positive cells in the testicular tissue. The percentage of positive cell area (Area%) was calculated.

### 2.7. Transmission Electron Microscopy of Testicular Tissue

Testicular samples were cut into small pieces and fixed with 2.5% glutaraldehyde at 4 °C. After resin embedding and polymerization, ultrathin sections were prepared and stained to observe the ultrastructure of the BTB by transmission electron microscopy (JEOL, Tokyo, Japan). This structure is composed of tight junctions between neighboring sertoli cells, which divide the seminiferous epithelium into distinct basal and adluminal compartments [35].

### 2.8. Immunofluorescence of Testicular Tissue

Testicular tissue sections were subjected to high-pressure antigen retrieval, washed with PBS, and blocked for 1 h. After blocking, the sections were incubated overnight at 4 °C with anti-ZO-1 (GB15195, 1:150, ServiceBio, Wuhan, China), anti-N-CADHERIN (T55015M, 1:200, Abmart, Shanghai, China), and anti-OCCLUDIN (GB151401, 1:150, ServiceBio, Wuhan, China), and anti-Connexin 43 (CX43) (GB11234, 1:150, ServiceBio, Wuhan, China) antibodies. After incubation, incubate with a secondary antibody homologous to the primary antibody at room temperature. Perform fluorescent staining using a TSA fluorescence kit, then observe and photograph the sections under a fluorescence microscope. Immunofluorescence intensity of target proteins within germ cells was quantified by Image J software (version 1.54g, NIH, Bethesda, MD, USA) under 400× microscopic fields. The mean fluorescence intensity was measured, and intergroup comparisons were presented as mean ± standard deviation (SD) [36].

### 2.9. Total RNA Extraction and RNA-Seq Analysis

Total RNA was extracted using the Trizol method from testicular tissue samples obtained from three mice in each of the 0 and 5 mg/kg/day dose groups (random sampling was performed from 10 experimental animals per group). RNA quality and purity were checked with an Agilent 2100 Bioanalyzer and the RNA 6000 Nano Lab-on-a-Chip Kit (5067-1511, Agilent, Santa Clara, CA, USA,). A cDNA library was built by pooling RNA from the testicular samples and then sequenced on the Illumina Novaseq™ 6000 platform. Raw reads were sorted and filtered to get clean reads for data analysis. We also calculated Q20, Q30, GC content, and clean read reproducibility. All later analyses were based on high-quality clean reads. The transcriptomes from the testicular samples were merged into a single transcriptome using gffcompare (version 0.9.8).

### 2.10. Differential Expression Analysis and Pathway Enrichment

Differentially expressed genes (DEGs) were screened, followed by GO and KEGG enrichment analyses. Differential expression analysis was performed using DESeq2 and edgeR, with DEGs defined as those exhibiting a false discovery rate (FDR) < 0.05 and an absolute fold change ≥ 2. Subsequently, GO functional annotation and KEGG pathway enrichment analysis were conducted on the identified DEGs using the DAVID tool, with a significance threshold of *p* < 0.05.

### 2.11. Cell Culture

This experiment utilized a mouse testicular sertoli cell line (TM4) obtained from the ATCC cell bank in the United States. Cells were maintained in DMEM/F12 medium supplemented with 10% FBS at 37 °C with 5% CO_2_. Based on this, the cells were exposed to 0, 100, 200, and 400 μM PFOA for 24 h. To investigate the effects of lactate, TM4 cells were first treated with sodium lactate (Nala) at concentrations of 0, 10, 20, and 30 mM. Subsequently, Nala (30 mM) was combined with PFOA (400 μM) for a 24 h co-treatment.

### 2.12. Cell Viability Assay

The cell viability was determined using the Cell Counting Kit-8 (CCK-8) assay kit. Concisely, the CCK-8 reagent was added following the treatment, and the absorbance values were measured using an instrument [37].

### 2.13. Lactic Acid Content Testing

After sonication of testicular tissue and treated TM4 cell samples, centrifuge to collect the supernatant; prepare the reagents were prepared as described in the lactate assay kit (SolarBio, Beijing, China, BC2235), add the reagents to the supernatant, incubate in the dark, measure the absorbance at 570 nm, and calculate the lactate concentration.

### 2.14. ATP Content Testing

After removing the old culture medium, 200 μL of ATP lysis buffer was added to each well. Cells were collected by pipetting, transferred to Eppendorf tubes, and centrifuged. The supernatant was used for analysis. ATP working solution was prepared using the ATP assay kit (Beyotime, Shanghai, China, S0027) with a reagent-to-diluent ratio of 1:9. To remove background ATP, 100 μL of working solution was added to each well, followed by 20 μL of sample or standard. Relative light unit (RLU) values were measured with a SpectraMax M2 luminometer reader, and ATP concentration was calculated.

### 2.15. RT-qPCR Testing

Total RNA was extracted from testicular tissue and PFOA-treated TM4 cells, and reverse transcription and qPCR were performed as previously described [12]. Relative mRNA expression was calculated using the 2^−ΔΔCt^ method with *Actb*, the internal control. Primer sequences are listed in Table 2.

### 2.16. Western Blot Assay

Samples were fully lysed in ice-cold RIPA buffer supplemented with PMSF protease inhibitor. After incubation on ice for 30 min, complete total protein extraction was achieved, which effectively prevented the degradation of target proteins. Proteins were extracted using RIPA lysis buffer and PMSF. Protein denaturation was carried out in accordance with the methods described in previous studies [38]. Following the preparation of SDS-PAGE gels based on the molecular weight of the target protein, and completion of electrophoresis, the proteins were transferred to a PVDF membrane. The membrane was incubated overnight at 4 °C with primary antibodies: anti-ZO-1 (GB15195, 1:1000, ServiceBio, Wuhan, China), anti-N-CADHERIN (T55015M, 1:1000, Abmart, Shanghai, China), anti-OCCLUDIN (GB151401, 1:1000, ServiceBio, Wuhan, China), anti-CX43 (GB11234, 1:1000, ServiceBio, Wuhan, China), anti-Hexokinase 1 (HK1, EC2.7.1.1) (T55530, 1:1000, Abmart, Shanghai, China), anti-Glucose Transporter 1 (GLUT1) (T55360, 1:1000, Abmart, Shanghai, China), anti-Lactate Dehydrogenase A (LDHA, EC 1.1.1.27) (T55348, 1:1000, Abmart, Shanghai, China), anti-Pan-Kla (PTM-1401RM, 1:1000, PTM Biolabs, Hangzhou, China), anti-H3K18la (PTM-1427RM, 1:1000, PTM Biolabs, Hangzhou, China), Glyceraldehyde-3-Phosphate Dehydrogenase (GAPDH) (A19056, 1:10,000, Abclonal, Wuhan, China), and anti-β-ACTIN (AC038, 1:100,000, Abclonal, Wuhan, China). After washing, membranes were incubated with the corresponding secondary antibody (A0208, 1:1000, Beyotime, Shanghai, China) for 1 h at room temperature. Blots were developed in the dark using Image Lab software (version 6.1.0), and quantitative analysis was performed with ImageJ software (version 1.54g, NIH, Bethesda, MD, USA).

### 2.17. ChIP-qPCR

ChIP-qPCR was performed as previously described with minor modifications [29]. Briefly, TM4 cells were cross-linked with formaldehyde, and chromatin was fragmented according to the manufacturer’s instructions (Beyotime, Shanghai, China, P2083S). After incubation with H3K18la antibody (PTM-1427RM, 6 μg/5 × 10^6^ cells, PTM Biolabs, Hangzhou, China) at 4 °C overnight, Protein A/G magnetic beads were added for 1 h incubation, followed by washing, chromatin elution, cross-link reversal, and spin column-based DNA extraction. Target DNA fragment enrichment was detected by RT-qPCR, with primers targeting specific H3K18la binding regions (Table 3).

### 2.18. Statistical Analysis

Statistical data were processed using SPSS 27.0.1, and figures were created with GraphPad Prism 10. All data are shown as mean ± SD. One-way ANOVA was used for intergroup comparisons, with pairwise comparisons assessed by LSD-t test. A significance level of *p* < 0.05 was applied.

## 3. Results

### 3.1. Exposure to PFOA Leads to Reduced Semen Quality and Impaired BTB Integrity in Male Mice

**Figure 1:**
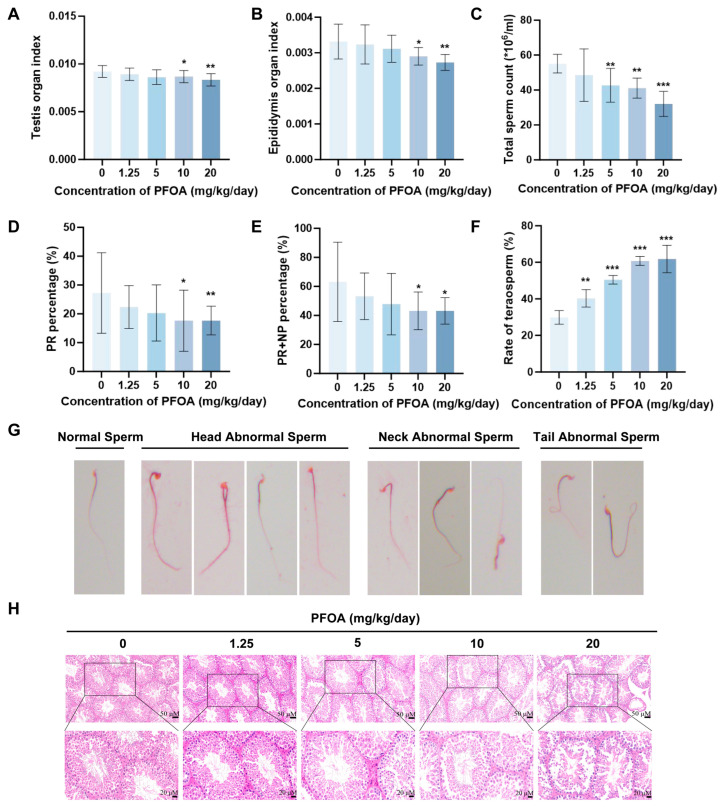
PFOA-induced testicular damage and sperm abnormalities. Increasing doses of PFOA exposure led to a reduction in both testicular (**A**) and epididymal (**B**) coefficients (*n* = 10), impaired sperm quality as evidenced by decreased sperm count (**C**), reduced PR% (**D**) and PR% + NP% (**E**), as well as an increased sperm abnormality rate (**F**) (*n* = 3); see (**G**) for major abnormal sperm morphologies, and induced histopathological damage to the testicular tissue (**H**) in mice (with a scale bar of 50 and 20 μM). (* *p* < 0.05; ** *p* < 0.01; *** *p* < 0.001, * vs. control group).

**Figure A1:**
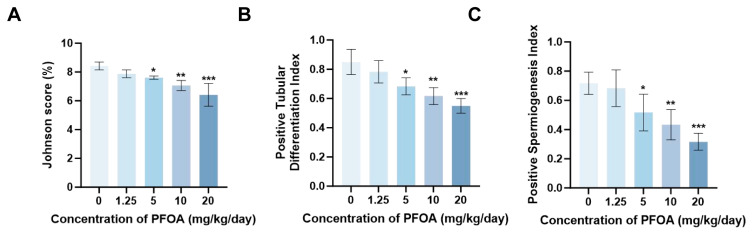
PFOA exposure caused histopathological damage to mouse testicular tissue, and further quantitative analysis using the (**A**) Johnson score, (**B**) the percentage of positive tubular differentiation index (TDI), and (**C**) the percentage of positive tubular spermiogenesis index indicated that spermatogenic function was impaired by PFOA. (*n* = 3, * *p* < 0.05; ** *p* < 0.01; *** *p* < 0.001, * vs. control group).

To clarify the severe detrimental effects of PFOA on the male mouse reproductive system, we first detected and calculated the testis/epididymis organ index of mice after PFOA exposure. Results indicated that testis/epididymis organ index in the 10 and 20 mg/kg/day groups showed a declining trend and was statistically significant (*p* < 0.05, Figure 1A,B). To determine whether PFOA impairs male fertility, we evaluated core indicators of male reproductive function, including sperm count and semen quality. The results demonstrated that high-dose PFOA was found to reduce sperm count (*p* < 0.05, Figure 1C), and the percentage of progressive motility (PR%) and the total motility percentage (the sum of the percentage of progressive motility and the percentage of non-progressive motility, PR% + NP%) were both significantly lower in the 10 and 20 mg/kg/day group (*p* < 0.05; Figure 1D,E). Statistical analysis of Abnormal sperm revealed that the rate of abnormal sperm was influenced by PFOA exposure and increased in a dosage-dependent manner with rising PFOA doses (*p* < 0.05, Figure 1F). The main types of abnormal sperm included head abnormal sperm (bulbous head, double head, hookless head, banana-shaped head), neck abnormal sperm (bent neck, neck enlargement) and tail abnormal sperm (bent tail) (Figure 1G). Pathological analysis of testicular tissue revealed that the normal testicular seminiferous tubules were structurally intact, with spermatogenic cells arrayed sequentially at all stages, and a large amount of sperm visible in the center of the seminiferous tubules. Compared with the control group, the arrangement of spermatogenic cells in the 1.25 and 5 mg/kg/day groups was slightly disordered, but no notable change was detected in the number of spermatogenic cell layers; whereas in the 10 and 20 mg/kg/day groups, the arrangement of spermatogenic cells was markedly loose and disordered, the number of spermatogenic cell layers was significantly reduced, and desquamation of spermatogenic epithelial cells was observed (Figure 1H). To better quantify histopathological changes, testicular H&E-stained sections were further evaluated using the Johnsen scoring system (*p* < 0.05, Figure A1A), TDI (*p* < 0.05, Figure A1B), and SPI (*p* < 0.05, Figure A1C). All three quantitative indices were significantly decreased, indicating a severe impairment of testicular histopathology and spermatogenic function in mice. In conclusion, PFOA disrupts spermatogenesis and induces testicular tissue damage.

**Figure 2:**
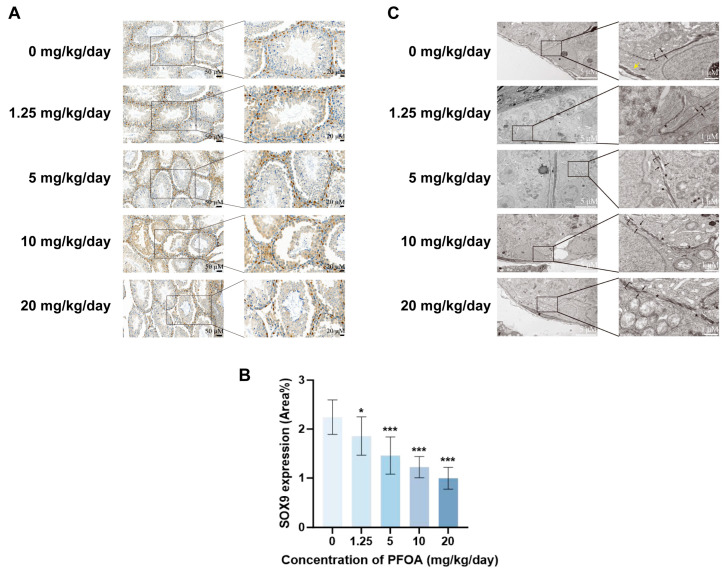
PFOA induces Sertoli cell reduction and blood-testis barrier damage. Immunohistochemical staining (with a scale bar of 50 and 20 μM. Meanwhile, brown indicates SOX9-positive cells) of the (**A**) SOX9 revealed that the (**B**) positive area ratio decreased gradually with the increase in PFOA exposure dose (*n* = 6). Meanwhile, transmission electron microscopy observations demonstrated obvious damage to the BTB ultrastructure (**C**); “black brackets and “black arrows”: tight junctions; “yellow arrows”: basal lamina of the seminiferous tubules; “black asterisks”: blurred or absent tight junctions, with a scale bar of 5 and 1 μM. (* *p* < 0.05; *** *p* < 0.001, * vs. control group).

**Figure 3:**
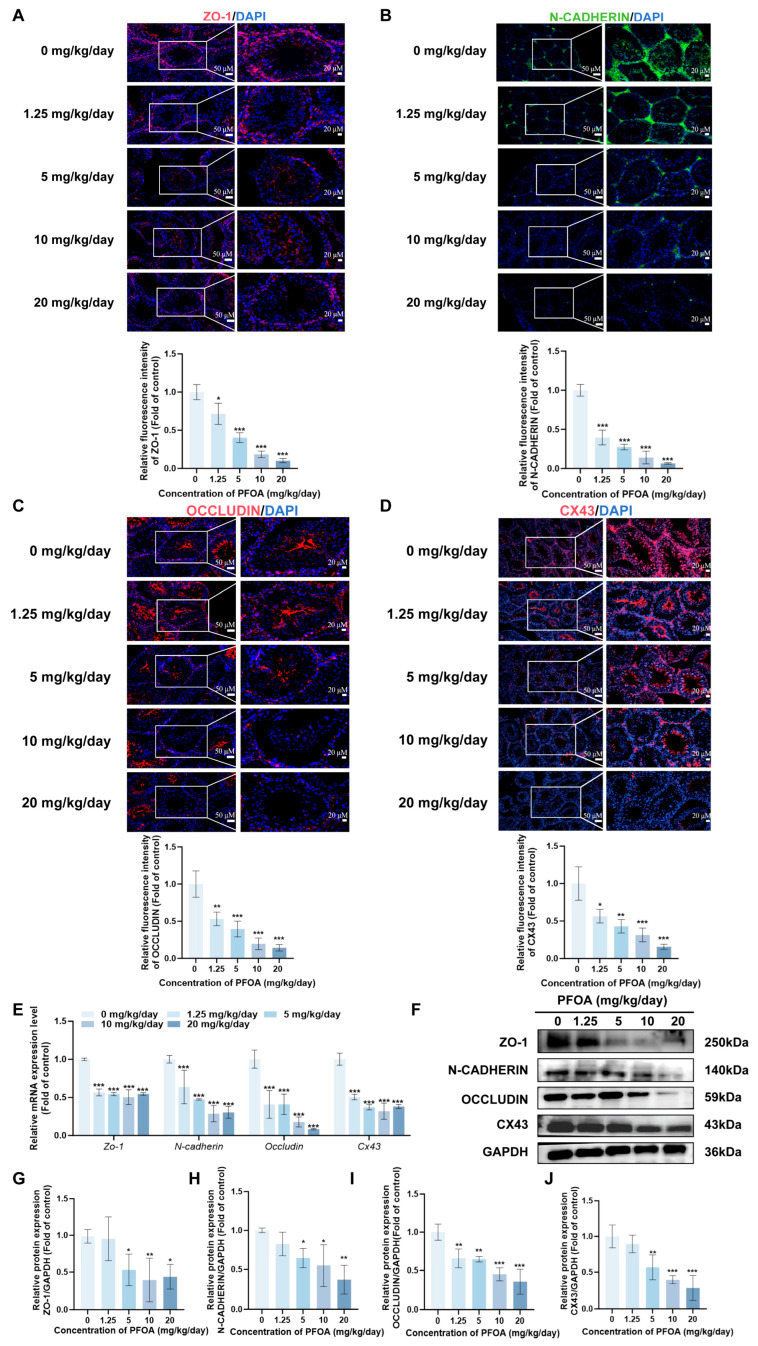
PFOA downregulates key BTB molecules. (**A**–**D**) Immunofluorescence staining of ZO-1 (**A**), N-CADHERIN (**B**), OCCLUDIN (**C**), and CX43 (**D**) in mouse testicular sections, with a scale bar of 50 and 20 μM. Among them, ZO-1, OCCLUDIN, and CX43 are marked in red, N-CADHERIN in green, and DAPI in blue. Fluorescence intensity decreases with increasing PFOA doses. (**E**) Relative mRNA expression levels of the four molecules, quantified by RT-qPCR, showing significant downregulation. (**F**) Western blot analysis of protein expression for ZO-1, N-CADHERIN, OCCLUDIN, and CX43 (**G**–**J**). Quantification of Western blot bands for ZO-1 (**G**), N-CADHERIN (**H**), OCCLUDIN (**I**), and CX43 (**J**) (*n* = 3, * *p* < 0.05; ** *p* < 0.01; *** *p* < 0.001, * vs. control group).

Sertoli cells form the scaffold of the spermatogenic epithelium, providing attachment and physical support for germ cells. Labeling (Figure 2A) and quantitative analysis of SOX9, a marker specific to sertoli cells, revealed that the proportion of SOX9-positive area declined in a dose-dependent pattern as increasing PFOA exposure (*p* < 0.05, Figure 2B). Transmission electron microscopy revealed the structure of BTB tight junctions. In the control group, the TJs exhibited a continuous, dense structure; however, as the PFOA dose increased, the integrity of the BTB was disrupted, with the tight junction structure becoming indistinct and showing localized cleavage, as well as multiple instances of discontinuity, loss or rupture (Figure 2C). To further verify the effects of PFOA on the BTB, the key BTB-related molecules including ZO-1, N-CADHERIN, OCCLUDIN and CX43 were detected. Immunofluorescence staining and quantitative analysis revealed that the relative fluorescence intensity of these molecules was significantly decreased in a PFOA dose-dependent manner (*p* < 0.05, Figure 3A–D). Western blot and RT-qPCR results further confirmed that both protein and mRNA expression levels of the four molecules were markedly inhibited (*p* < 0.05, Figure 3E–J).

### 3.2. Transcriptome Sequencing Reveals Differentially Expressed Genes Associated with Glycolysis Metabolism Downregulated by PFOA Exposure

**Figure 4:**
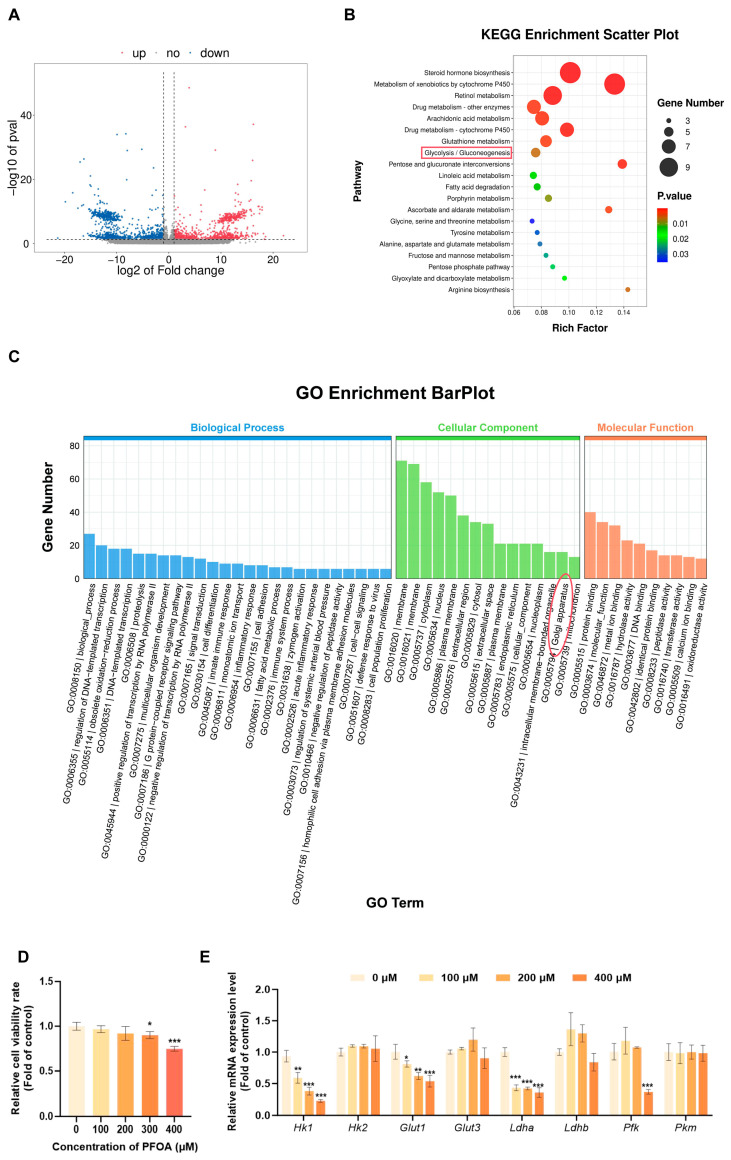
PFOA altered the expression of glycolysis-related genes. Based on RNA-seq analysis, the total RNA expression profiles of testicular tissues in the control and 5 mg/kg/day PFOA-exposed groups were compared (**A**). KEGG pathway enrichment (**B**) and GO functional enrichment analyses (**C**) of differentially expressed genes revealed that the glycolysis pathway served as a key target of PFOA. After 24 h of PFOA treatment in TM4 cells, cell viability was decreased (**D**), accompanied by significant changes in the expression of glycolysis-related genes (**E**). (*n* = 3, * *p* < 0.05; ** *p* < 0.01; *** *p* < 0.001, * vs. control group).

To elucidate the regulatory role of PFOA exposure on glycolysis metabolism in male reproductive toxicity, this study first analyzed the gene expression profiles of testicular tissue from control mice and those exposed to 5 mg/kg/day PFOA using transcriptomic sequencing. The results revealed significant differentially expressed genes between the two groups (Figure 4A). Subsequent GO and KEGG pathway enrichment analyses of the dysregulated genes indicated that the glycolysis pathway was one of the significantly enriched key pathways (Figure 4B,C). Given that sertoli cells synthesize lactate via the glycolysis pathway, providing a key energy substrate for spermatogenesis, this study will focus on the glycolysis pathway in subsequent research. Firstly, the CCK-8 cell viability assay was used to assess the effect of PFOA exposure for 24 h on the viability of TM4 cells. The study found that, relative to the control group, treatment with 100 μM PFOA had no remarkable effect on cell viability; cell viability showed a downward trend following treatment with 200 μM, but this discrepancy is not remarkably meaningful; whereas cell viability was remarkably reduced in both the 300 μM and 400 μM treatment groups (*p* < 0.05, Figure 4D). Based on the above results, this study selected 100, 200 and 400 μM as the PFOA treatment concentrations for subsequent experiments. Subsequently, to validate the glycolysis pathway identified by transcriptomic analysis, transcription levels of crucial genes in the glycolysis pathway were assessed using RT-qPCR. It was found that, among the key glycolysis genes examined, the expression of *Hk1*, *Glut1* and *Ldha* was significantly downregulated in all dose groups, whilst Phosphofructokinase (*Pfk*) was remarkably downregulated only in the high-dose exposure group; no remarkable changes were observed in the remaining genes (*p* < 0.05, Figure 4E). In summary, PFOA may interfere with spermatogenesis by inhibiting glycolysis in sertoli cells.

### 3.3. Exposure to PFOA Downregulates the Expression of Critical Glycolysis Enzymes and Induces Dysfunction of the BTB

**Figure 5:**
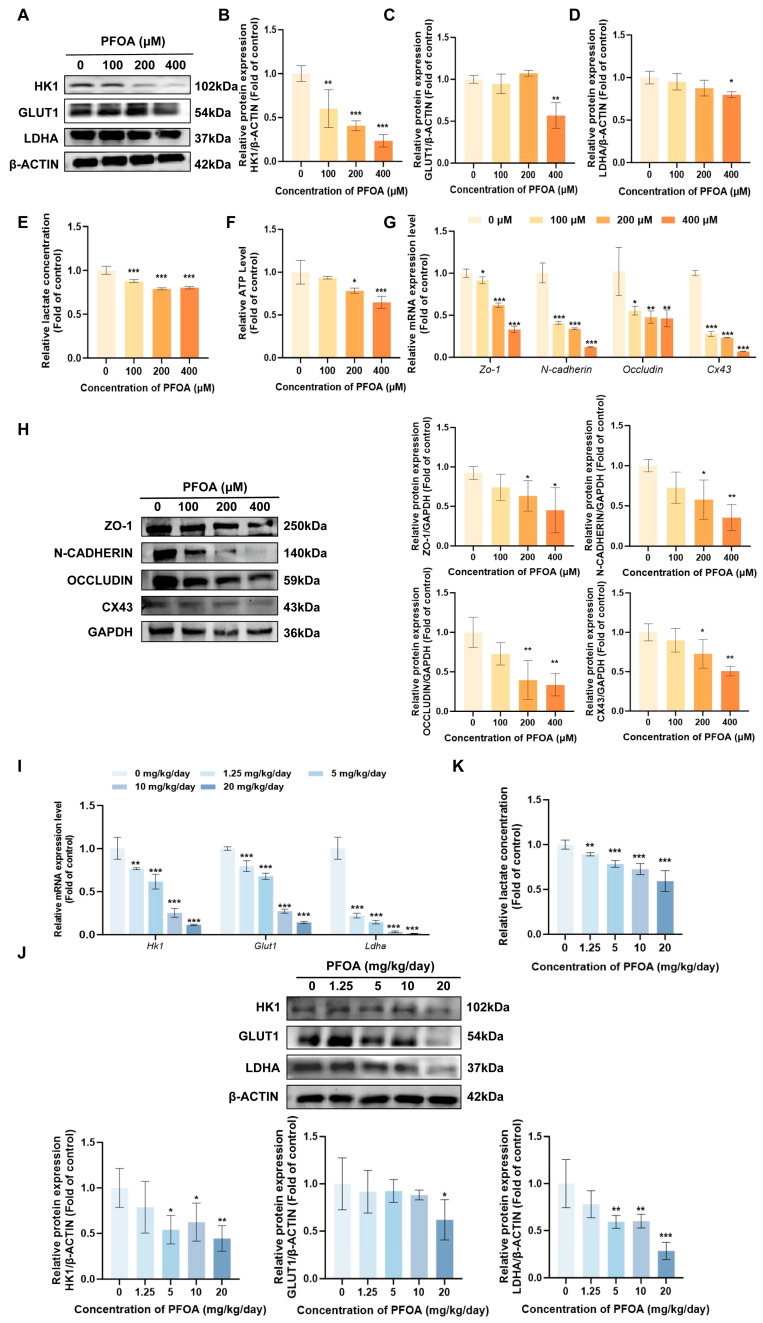
PFOA inhibited the expression of glycolysis and BTB-related molecules in TM4 cells and mouse testicular tissues. In PFOA-treated TM4 cells, the protein levels of key glycolytic molecules, including HK1 (**B**), GLUT1 (**C**) and LDHA (**D**), were downregulated (**A**–**D**), accompanied by reduced production of lactate (**E**) and ATP (**F**). Meanwhile, both the gene (**G**) and protein (**H**) expression of BTB-related factors were decreased in TM4 cells. Consistent with the cellular results, the mRNA (**I**) and protein (**J**) levels of glycolysis-related molecules were also suppressed in mouse testis, along with a decline in lactate concentration (**K**). (*n* = 3, * *p* < 0.05; ** *p* < 0.01; *** *p* < 0.001, * vs. control group).

Based on transcriptomic enrichment analysis suggesting alterations in the glycolysis pathway following PFOA exposure, this study further validated the key molecules of this pathway in TM4 cells. Western blot results showed that HK1 GLUT1 and LDHA protein levels exhibited a downward trend (*p* < 0.05, Figure 5A–D). To further verify whether the inhibition of glycolysis in sertoli cells affects energy metabolism, the levels of lactic acid and ATP in TM4 cells were detected, and both were significantly decreased (*p* < 0.05, Figure 5E,F). It is evident from the above results that PFOA may interfere with lactate production by regulating the expression of key glycolysis molecules, namely HK1, GLUT1 and LDHA. Meanwhile, after 24 h of PFOA exposure, the mRNA and protein levels of ZO-1, N-CADHERIN, OCCLUDIN and CX43 in TM4 cells decreased with increasing PFOA concentrations (*p* < 0.05; Figure 5G,H). It suggests that exposure to PFOA can simultaneously induce abnormalities in glycolysis in TM4 cells and damage to the BTB. At the animal level, to verify whether PFOA causes abnormalities in glucose metabolism in testicular tissue, analyses by RT-qPCR and Western blot revealed that the gene and protein expression levels of HK1, GLUT1 and LDHA were profoundly attenuated in testicular tissue (*p* < 0.05, Figure 5I,J), and lactate production was also significantly reduced (*p* < 0.05, Figure 5K), further supporting the inhibitory effect of PFOA on glucose metabolism in testicular tissue. In summary, PFOA inhibits the expression and function of crucial molecules involved in glycolysis in testes and TM4 cells, accompanied by damage to the BTB; this suggests that glycolytic dysfunction may be involved in the process of PFOA-induced male reproductive toxicity.

### 3.4. The Role of PFOA-Mediated Downregulation of H3K18la Modification in the Inhibition of Glycolysis and Damage to the BTB

**Figure 6:**
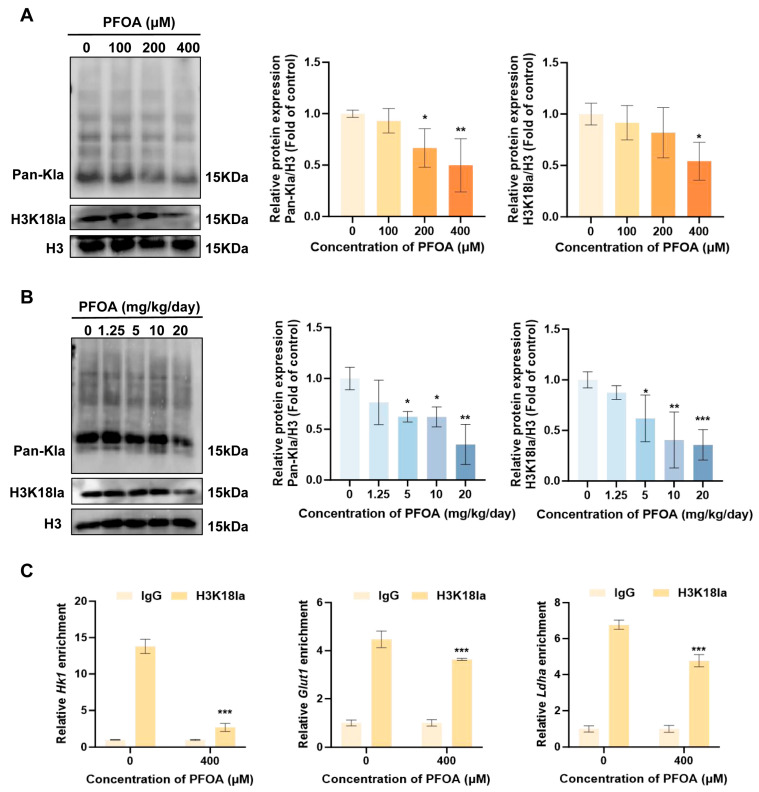
Effects of PFOA on H3K18 lactylation and the transcriptional regulation of key genes involved in glycolysis. PFOA exposure reduced the modification levels of Pan-Kla and H3K18la in TM4 cells (**A**) and mouse testicular tissues (**B**). ChIP-qPCR results further demonstrated that PFOA altered the binding of H3K18la to glycolytic genes, thereby regulating their transcription (**C**). (*n* = 3, * *p* < 0.05; ** *p* < 0.01; *** *p* < 0.001, * vs. control group).

**Figure 7:**
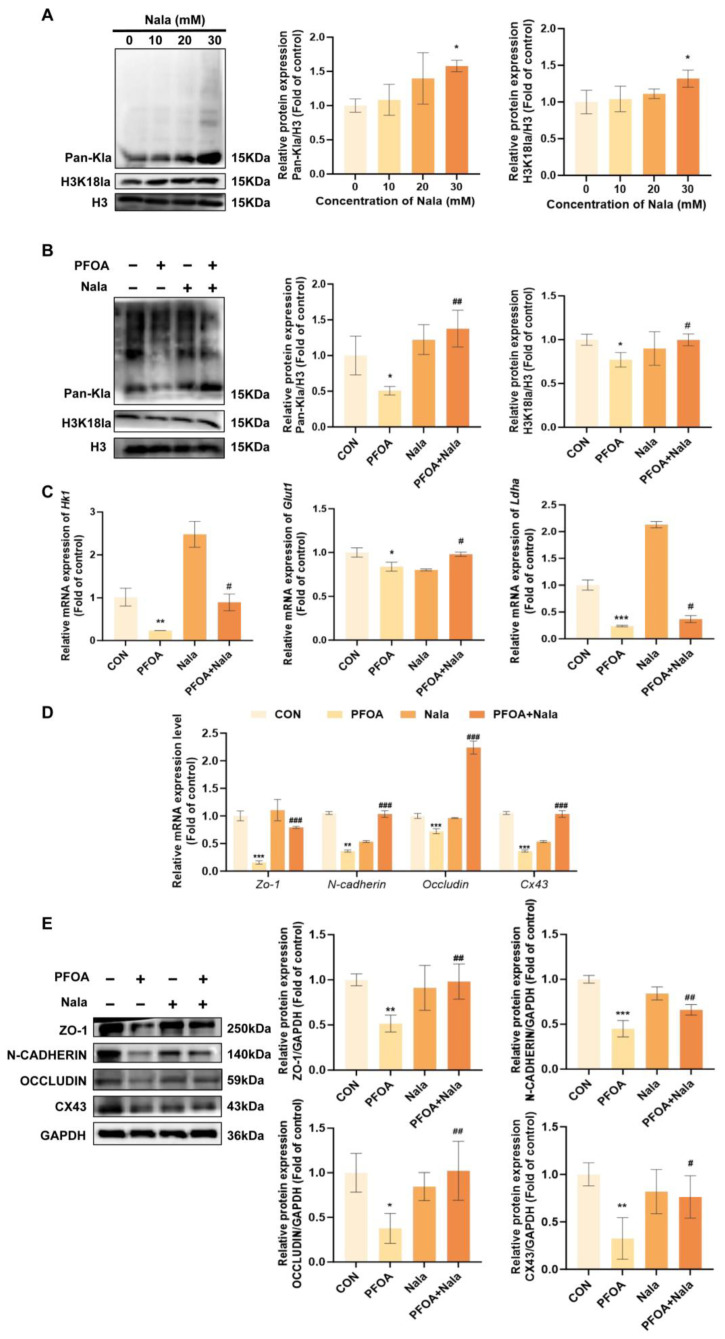
Protective effects of Nala against PFOA-induced reproductive damage. Gradient Nala treatment for 24 h significantly elevated the levels of Pan-Kla and H3K18la in TM4 cells (**A**). After co-treatment with 30 mM Nala and 400 μM PFOA for 24 h, the PFOA-mediated reductions in Pan-Kla and H3K18la were notably restored (**B**). The decreased expression of Hk1, Glut1 and Ldha was partially rescued (**C**), and the downregulated mRNA (**D**) and protein (**E**) levels of BTB-related molecules were also markedly recovered. (*n* = 3, * *p* < 0.05; ** *p* < 0.01; *** *p* < 0.001; * vs. control group. # *p* < 0.05; ## *p* < 0.01; ### *p* < 0.001; # vs. PFOA group).

Given that lactate serves as the primary substrate for lactylation and that changes in its levels may influence the lactylation status of proteins, This study further investigations the putative regulatory function of PFOA in lactylation. First, changes in total lactylation and H3K18la levels were assessed in testes and TM4 cells. The results illustrated that following PFOA exposure, total lactylation levels were remarkably reduced in both testes and TM4 cells, and H3K18la levels decreased in a dose-dependent manner (*p* < 0.05, Figure 6A,B). Previously conducted research has revealed that H3K18la accumulates in the promoter regions of genes associated with glycolysis and plays a role in their transcriptional regulation [39]. On this basis, this study further confirms the transcriptional regulatory contribution of H3K18la on *Hk1*, *Glut1*, and *Ldha*. Using ChIP-qPCR technology, for the aforementioned genes, specific primers were designed for the promoter regions, and their enrichment levels were assessed using the H3K18la antibody. The results indicate that PFOA exposure remarkably suppressed the enrichment of H3K18la in the promoter regions of the *Hk1*, *Glut1*, and *Ldha* genes (*p* < 0.05, Figure 6C). Next, to elucidate the molecular mechanism by which PFOA regulates glycolysis via lactylation, this study first subjected TM4 cells to gradient treatments with 10, 20 and 30 mM Nala. With the increase in Nala dosage, Pan-Kla and H3K18la modifications in TM4 cells were markedly elevated. (*p* < 0.05, Figure 7A). This confirms that lactate content regulates lactylation modification. Building on these findings, when 400 μM PFOA was co-administered with 30 mM Nala in TM4 cells for 24 h, it was found that exogenous supplementation with Nala significantly reversed the PFOA-induced downregulation of Pan-Kla and H3K18la levels (*p* < 0.05, Figure 7B). The downregulation of mRNA expression in the key glycolytic genes *Hk1*, *Glut1* and *Ldha* induced by PFOA was also significantly reversed by Nala (*p* < 0.05, Figure 7C). The above results reveal that PFOA regulates the transcriptional expression of genes associated with glycolysis by reducing the level of H3K18la modification. In addition, this study indicate that Nala treatment partially restored the downregulation of BTB key molecules—ZO-1, N-CADHERIN, OCCLUDIN and CX43—by PFOA at the transcriptional and translational levels (*p* < 0.05; Figure 7D,E). In summary, this study confirms that PFOA inhibits the glycolytic pathway by reducing the level of H3K18la modification, thereby causing damage to the BTB. Meanwhile, Nala intervention can effectively alleviate PFOA-induced BTB damage by restoring lactylation modification and glycolytic function.

## 4. Discussion

As a typical environmental endocrine disruptor, PFOA causes reproductive toxicity, organ damage, and metabolic disorders through various mechanisms, posing a serious threat to human health. Before PFOA was subject to strict regulation, serum PFOA concentrations in workers in relevant occupations reached 114.1 μg/mL [40]. Such high levels coupled with the fact that the half-life of this pollutant in the human body can be as long as 3.8–5.1 years [41,42], makes the potential health risks arising from long-term accumulation a particular concern.

Based on existing male reproductive toxicity studies, this study selected four PFOA exposure doses (1.25, 5, 10, and 20 mg/kg/day) to systematically evaluate the dose-effect relationship, among which 1.25 mg/kg/day, as the threshold dose simulating environmental exposure [43], results in a mouse serum PFOA concentration of approximately 25 μg/mL [35], consistent with that of the general population in highly polluted areas [6], and was set as the lowest exposure group to reflect the potential risks of long-term low-dose exposure; the two medium-high doses of 5 and 10 mg/kg/day can induce significant reproductive damage [44,45]. 20 mg/kg/day is widely used as a positive control, and continuous 28 days exposure can cause typical reproductive toxicity in mice (reduced sperm count, decreased testosterone levels, and seminiferous tubule damage) [46], with a serum PFOA concentration of up to 105 μg/mL [40,44]. Given that the elimination half-life of PFOA is considerably longer in humans (3.8–5.1 years) than in mice (15–20 days) [41,42], this significant interspecies difference in toxicokinetics necessitates higher daily doses in mice to achieve a systemic PFOA burden comparable to that observed in occupationally exposed humans. Therefore, the 20 mg/kg/day high dose used in this study provides a toxicologically sound simulation of the high internal exposure levels seen in occupational settings. In summary, the selected dose range of 1.25–20 mg/kg/day offers a robust and well-justified experimental basis for systematically elucidating the dose–response relationship between PFOA exposure and male reproductive toxicity.

Epidemiological studies have confirmed that serum PFOA levels are positively correlated with PFOA accumulation in testicular tissue, with median concentrations reaching 1.7 ng/g and sperm motility being significantly reduced at the same time [47,48]. PFOA affects testicular structure, function, and development by interfering with the secretion of male sex hormones [49,50]. Research on animals has further demonstrated that exposure to PFOA can reduce sperm motility and increase the rate of sperm abnormalities [51,52,53]. The results obtained in the present study closely reflect those reported in the above-mentioned literature: when male mice were exposed to PFOA for 35 days, testicular and epididymal indices decreased as the PFOA dose increased, and pathological symptoms gradually worsened. Specifically, these manifested as a notable reduction in the number of germ cells within the seminiferous tubules, disordered organization of germ cells, and a decrease in the number of sperm cells in the central region of the seminiferous tubules. The results of sperm analysis revealed that the group exposed to PFOA exhibited a reduction in sperm count (5 mg/kg/day) and motility (10 mg/kg/day), whereas more pronounced damage was detected in the high-dose group. Furthermore, the rate of sperm abnormalities showed an upward trend as the PFOA dose increased. These findings indicate that PFOA has a clear dose-dependent toxic effect on the male reproductive system, and that this toxicity can manifest at environmentally relevant levels of exposure.

In this study, PFOA was shown to disrupt the BTB integrity, as evidenced by a significant reduction in the expression of key BTB molecules—including the tight junction proteins ZO-1 and OCCLUDIN, the gap junction protein CX43, and the adhesion molecule N-CADHERIN—within testicular tissue and TM4 cells, both at the transcriptional and protein levels. Immunofluorescence staining revealed a considerably diminished fluorescence intensity in these proteins, whereas transmission electron microscopy revealed blurred TJs and localized disruption within the BTB ultrastructure, along with multiple instances of discontinuity, loss, and rupture. These observations align with previous findings [54,55,56] suggesting that PFOA disrupts the integrity of the BTB, thereby affecting spermatogenesis. Following RNA sequencing analysis, genes with differential expression were identified in the testes of control mice compared to those exposed to 5 mg/kg/day PFOA. Using GO and KEGG pathway enrichment analyses, researchers identified the glycolysis pathway as a significant target in male reproductive damage caused by PFOA.

Further in vitro and in vivo validation experiments indicated that PFOA exposure can induce glycolysis dysfunction, primarily manifested as significant downregulation of the crucial glycolytic enzymes HK1, GLUT1 and LDHA, leading to reduced levels of lactate and ATP. In sertoli cells—LDHA and LDHB are the primary expressed isoforms. Importantly, LDHA-dependent lactate production in sertoli cells has been demonstrated to be a critical regulator of spermat-ogenesis, particularly during the spermiogenesis stage [27,57,58,59]. Consistently, Marinaro [60] shows that environmental pollutants trigger oxidative stress in sperm, which in turn alters protamine conformation, reduces its DNA-binding ability, and leads to sperm DNA damage. These findings are important and provide a complementary perspective focusing on downstream mechanisms for our own work. Based on these findings, we propose that disruption of the BTB creates a pro-oxidative state within the seminiferous epithelium, rendering developing spermatids more vulnerable to oxidative insult.

Recent studies have confirmed that lactic acid not only serves as a substrate for energy metabolism but is also involved as a signaling molecule in epigenetic regulation, with its levels directly influencing the extent of histone lactylation [61,62]. As a typical histone lactylation, H3K18la is mainly studied in the domain of oncology; however, in recent years, its association with male reproduction has gradually attracted attention [63,64]. This is the first study to examine H3K18la in PFOA-induced BTB damage. The results show that PFOA significantly suppresses the levels of Pan-Kla and H3K18la in testicular tissue and TM4 cells. Under physiological conditions, H3K18la can be recruited to the promoter regions of specific genes, directly driving their transcription. Previous studies have reported that HK-mediated enhancement of glycolysis can lead to lactic acid accumulation, while H3K18la is enriched in the *Hk2* promoter region at the same time [39]. In this study, ChIP-qPCR experiments demonstrated that H3K18la is enriched in the promoter regions of the *Hk1*, *Glut1*, and *Ldha* genes and is significantly reduced following PFOA exposure, indicating that PFOA suppresses the transcriptional expression of key glycolysis genes by reducing the level of H3K18la. To further test this hypothesis, TM4 cells were treated simultaneously with PFOA and Nala for 24 h. The results indicated that supplementation with Nala significantly reversed the PFOA-induced downregulation of lactylation levels (Pan-Kla and H3K18la), and also effectively rescued the downregulation of *Hk1*, *Glut1*, and *Ldha* expression. Furthermore, exogenous supplementation with lactic acid significantly alleviated the damage caused by PFOA to the BTB, as evidenced by the partial recovery of the expression of key BTB molecules. The above results clearly demonstrate that the architecture and function integrity of the BTB complex depends strongly on the energy supply from sertoli cells. When glycolysis is impaired and there is a shortage of lactate and ATP, the function of sertoli cells is inhibited, leading to a reduction in H3K18la levels, which in turn regulates the transcriptional expression of glycolysis-related genes, ultimately resulting in impaired BTB function and disrupted spermatogenesis. This mechanism reveals a novel pathway through which environmental pollutants induce male reproductive toxicity by disrupting the coupling between energy metabolism and epigenetic regulation.

This study innovatively introduces the metabolism-epigenetic coupling mechanism into environmental reproductive toxicology research, broadening the research system for reproductive toxicity induced by persistent organic pollutants. First, mechanistically, while previous studies have emphasized oxidative stress and endocrine disruption, we demonstrate that PFOA primarily targets sertoli cell glycolysis. Second, tissue-specifically, we show that PFOA directly disturbs testicular local metabolism, extending beyond its well-known effects on systemic glucose homeostasis. Third, epigenetically, we provide the first evidence linking PFOA exposure to H3K18 lactylation in male reproductive toxicity. Fourth, we test exogenous lactate as a feasible intervention, which has not been attempted previously.

However, the following limitations should be acknowledged: first at the molecular mechanistic level, although it has been demonstrated that H3K18la is enriched in the *Hk1*, *Glut1*, and *Ldha* promoter regions and participates in their transcriptional regulation, its specific binding sites and the downstream gene networks it regulates have not yet been identified. Furthermore, the interactive regulatory roles of H3K18la and other epigenetic modifications (such as methylation and glycosylation) in sertoli cells remain to be elucidated; second, with regard to the duration of exposure and intergenerational effects, this study only examined subacute exposure (35 days) and did not explore the chronic cumulative effects of long-term low-dose exposure. Simultaneously, the sample size for sperm morphology and RNA sequencing (*n* = 3) was not consistent with the sample size for other sperm- and organ-level endpoints (*n* = 10). Third, in terms of mechanistic research, this study primarily focused on the glycolysis pathway and did not further investigate whether PFOA forms synergistic networks with lactylation through other pathways such as the regulation of mitochondrial metabolism, oxidative stress or lipid metabolism. Finally, as this study used mice as a model, the suitability of these mechanistic findings to describe dynamics in humans requires further validation using epidemiological data and human biological samples.

## 5. Conclusions

This study demonstrated that exposure to PFOA can induce pathological damage to testicular tissue, significantly impair semen quality, and compromise the architecture and function integrity of the BTB. Further mechanistic studies have shown that PFOA reduces lactate production by impairing the transcription of critical enzymes involved in glycolysis in sertoli cells (HK1, GLUT1, LDHA), thereby downregulating the level of histone H3K18la modification. Furthermore, reduced H3K18la modification subsequently inhibits the transcriptional expression of glycolysis-related genes, forming a “glycolysis–lactate–H3K18la” chain. This study is the first to elucidate the molecular mechanisms underpinning PFOA-induced male reproductive toxicity from the perspective of the “synergistic interaction between metabolic reprogramming and epigenetic modifications”. It confirms the pivotal role of H3K18la in PFOA-induced damage to the BTB and provides a new potential target for intervening in reproductive dysfunction caused by PFOA exposure.

## Figures and Tables

**Table 1:** Johnsen Scoring Criteria of Seminiferous Tubules.

Histologic Criteria	Score
Fully completed and orderly spermatogenesis	10
Abundant spermatozoa with disorganized spermatogenic process	9
Limited quantity of mature spermatozoa	8
Numerous spermatids with absence of mature spermatozoa	7
Rare occurrence of immature spermatids	6
Abundant spermatocytes without spermatids or spermatozoa	5
Sparse distribution of spermatocytes	4
Sole presence of spermatogonia	3
Lack of all germ cell populations	2
Absence of germ cells and Sertoli cells	1

**Table 2:** RT-qPCR primer used in the current study.

Gene	Forward Sequence (5′–3′)	Reverse Sequence (5′–3′)
*Zo-1*	GTTGGTACGGTGCCCTGAAAGA	GCTGACAGGTAGGACAGACGAT
*N-cadherin*	CCTCCAGAGTTTACTGCCATGAC	CCACCACTGATTCTGTATGCCG
*Occludin*	TGGCAAGCGATCATACCCAGAG	CTGCCTGAAGTCATCCACACTC
*Cx43*	GGTGATGAACAGTCTGCCTTTCG	GTGAGCCAAGTACAGGAGTGTG
*Hk1*	GCTGAAGGATGACCAAGTCAA	AATCCCCCTTTTCTGAGCCG
*Hk2*	ATGATCGCCTGCTTATTCACG	CGCCTAGAAATCTCCAGAAGGG
*Glut1*	GCTTCTCCAACTGGACCTCAAAC	ACGAGGAGCACCGTGAAGATGA
*Glut3*	ATGGGGACAACGAAGGTGAC	GTCTCAGGTGCATTGATGACTC
*Ldha*	CGGGAGGGCAGCTTTCTAA	TCATCCGCCAAGTCCTTCATT
*Ldhb*	TTCTGCTCGATTCCGCTACC	ATGCCGTACATTCCCTGTCC
*Pkm*	CAGAGAAGGTCTCTCTGGCTCA	GCCACATCACTGCCTTCAGCAC
*Pfk*	AAGAGGAACAAGCAGTGGC	TTCCTCGGAGTTCCCC
*Actb*	TATAAAACCCGGCGGCGCA	TCATCCATGGCGAACTGGTG

**Table 3:** ChIP-qPCR primer used in the current study.

Gene	Forward Sequence (5′–3′)	Reverse Sequence (5′–3′)
*Hk1*	GCCAGTGCTCTAACTGCTGA	ACACATGCTGAGAGACCACG
*Glut1*	GTGACGATCTGAGCTACGGG	TTACTCACCTTGCTGCTGGG
*Ldha*	AGTGTGGGGGAGTAGCCTAG	CCGGACACCATCTTGCTTCT

## Data Availability

The data used to support the findings of this study are available from the corresponding author upon reasonable request.

## Appendix A

Figure A1. Quantitative histopathological analysis of testicular sections using three validated indices.
